# Multiresolution generalized *N* dimension PCA for ultrasound image denoising

**DOI:** 10.1186/1475-925X-13-112

**Published:** 2014-08-05

**Authors:** Danni Ai, Jian Yang, Yang Chen, Weijian Cong, Jingfan Fan, Yongtian Wang

**Affiliations:** 1 Beijing Engineering Research Center of Mixed Reality and Advanced Display, School of Optics and Electronics, Beijing Institute of Technology, Beijing 100081, China; 2 Key Laboratory of Computer Network and Information Integration, Ministry of Education of China, Laboratory of Image Science and Technology, Southeast University, Nanjing 210096, China

**Keywords:** Multiresolution, Multilinear subspace learning, Noise reduction

## Abstract

**Background:**

Ultrasound images are usually affected by speckle noise, which is a type of random multiplicative noise. Thus, reducing speckle and improving image visual quality are vital to obtaining better diagnosis.

**Method:**

In this paper, a novel noise reduction method for medical ultrasound images, called multiresolution generalized *N* dimension PCA (MR-GND-PCA), is presented. In this method, the Gaussian pyramid and multiscale image stacks on each level are built first. GND-PCA as a multilinear subspace learning method is used for denoising. Each level is combined to achieve the final denoised image based on Laplacian pyramids.

**Results:**

The proposed method is tested with synthetically speckled and real ultrasound images, and quality evaluation metrics, including MSE, SNR and PSNR, are used to evaluate its performance.

**Conclusion:**

Experimental results show that the proposed method achieved the lowest noise interference and improved image quality by reducing noise and preserving the structure. Our method is also robust for the image with a much higher level of speckle noise. For clinical images, the results show that MR-GND-PCA can reduce speckle and preserve resolvable details.

## Introduction

An accurate anatomical display of organs plays a very important role in current-day medical diagnosis. Among a diverse set of medical imaging modalities, the use of ultrasound has increased rapidly because of its non-invasive nature, hardware portability, inexpensiveness, and real-time imaging. However, ultrasound images have much more noise than other medical imaging modalities, such as computer tomography (CT) and magnetic resonance imaging (MRI). The primary source of ultrasound imaging noise is considered as speckle noise, which deteriorates image quality, degrades fine details and edge definition, and limits contrast resolution [1]. Consequently, reducing speckle noise while preserving anatomic information is necessary to reliably delineate the regions of interest and increase the diagnostic potential of ultrasounds.

Extensive work using various techniques has been conducted to reduce speckle noise. These techniques can be classified into two categories according to the moment when speckle reduction is produced, namely, compounding approach and post-processing approach [2]. Compounding approach modifies the data acquisition procedure to produce several images of the same region and then combine them to form a single image. This approach is beyond the scope of this paper. Post-processing approach processes ultrasound images after being generated. Most studies have focused on this approach, in which filtering methods are developed to be applied directly to the original image. Based on the assumption that speckle is essentially a multiplicative noise [3], filtering methods composed of many fixed and adaptive filters have been proposed, such as L2-mean filter [4], adaptive filter reduction [5], adaptive weighted median filter [6], nonlinear diffusion [7], Map estimation [8], and so on.

Although techniques for processing multiplicative noise exist, they are not as well developed as the techniques for additive noise. Generally, the multiplicative nature of speckle noise formation can be converted into an additive noise through the application of logarithmic transformation to a speckled image. Subsequently, various types of state-of-the-art denoising algorithms can be utilized for ultrasound despeckling. State-of-the-art algorithms are mainly divided into three categories: (1) algorithms applied in the frequency domain, (2) algorithms applied in the spatial domain, and (3) algorithms applied in both spatial and frequency domains. Wavelet-based filters implement denoising in the frequency domain, such as Bayesian least squares (BLS) Gaussian scald mixture (GSM) wavelet denoising method [9]. This method transforms the image into the wavelet domain, assumes the GSM model on neighborhoods, and denoises using BLS estimation. However, a rich amount of different local structures are contained in medical images, and these structures cannot be well represented by only one fixed wavelet basis. Thus, numerous visual artifacts can be introduced through wavelet-based methods. Using the temporal perspective, spatial denoising methods are proposed to discover redundancies in a single image, such as non-local mean-based methods [10,11]. These methods extract the commonalities and use a weighted average based on similarity. Recently, denoising methods implemented in both spatial and frequency domains, such as K-SVD method [12], non-local collaborative filtering [13], and principle component analysis (PCA)-based methods [14,15], have attracted attention. The core idea of these denoising methods is the use of sparser representation instead of a spatial weighted average [16,17].

Recent studies have shown that PCA-based methods can obtain impressive achievements. Bags of patches extracted from the noisy image are processed by PCA to reduce the redundancy between data. PCA is a linear subspace learning method that has been applied widely for dimensionality reduction by searching for the maximum variance directions. However, conventional PCA was originally proposed to process one-dimensional vectors, which require all input data to first be unfolded into a vector in order to fit the nature of the PCA. For similar patches extracted to reduce redundancy, vectorization destroys the structure of the input patches, resulting in the overfitting problem because the dimension of the vectorized data may be larger than that of the sample number.

This paper proposes a novel multiresolution generalized *N* dimension PCA for ultrasound image denoising called the MR-GND-PCA. The issue of noise for ultrasound images can be reduced with higher frequency imaging, but this would limit the depth of ultrasound penetration. Thus, the noise is decided by the different penetrations. An intuitive justification is that ultrasound images are equally likely to be viewed from different distances. Our proposed method assumes that the statistics of ultrasound images are invariant to changes in the spatial scale. Additionally, the larger scale structure is lost when the image is decomposed into small patches. This case can be avoided by employing the multiresolution/multiscale approach. Instead of traditional PCA, a multilinear subspace learning method called GND-PCA is also utilized to preserve useful information and noise suppression. The proposed method has three stages. First, a Gaussian pyramid for the input noisy image is built with a multiresolution scheme. The noise in the ultrasound image is gradually smoothed and computational time is reduced when computing in higher pyramid levels. The stacks are then built with multiscale images produced from each pyramid level. Second, dense patches (two-way array) with the same size are extracted, and similar patches are grouped together in three-way arrays in different stacks. GND-PCA is directly used to filter the three-dimensional array with its original structure in order to overcome the drawbacks of conventional PCA, as well as to consider the interrelationship among different modes of each patch to maintain useful information and remove noise. Denoised patches are then placed back into their original positions. Third, after obtaining the representative images in each stack, a combination motivated by Laplacian pyramids is obtained. And then, the denoising image is yielded from the combined image with exponential transformation and sharpen procedure.

The organization of the paper is as follows. Section Related work provides a brief introduction of related work. The proposed MR-GND-PCA denoising method is outlined in Section Methodology. The experimental design is presented and results are discussed in Section Experiments and results. Finally, Section Discussion and conclusions concludes the work.

## Related work

Generalized *N* dimension PCA (GND-PCA) is the main related work of our proposed method. For more details please refer to [18]. The GND-PCA is briefly presented as follows.

Traditional PCA [19] is one of the fundamental linear subspace learning techniques for data analysis. The main purpose of PCA is to reduce data dimension and retain information that characterizes the variation of data as much as possible. Thus, PCA can be widely used in noise reduction, i.e., to keep useful information and eliminate high-frequency noise. However, the data must be unfolded into one-dimensional vectors to fit with the PCA processing. For a two-dimensional image or *N*-way array, this unfolding process destroys the structure of the input data. PCA also suffers from a generalization problem because the calculated bases with PCA cannot represent data very well if the number of samples is much smaller than the dimension of the unfolded vector.

Xu et al. [18] proposed a multilinear subspace learning method called generalized *N* dimension PCA (GND-PCA) to overcome these insufficiencies. GND-PCA is proposed by extending the conventional linear PCA based on multilinear algebra. In multilinear algebra, higher-dimensional data are treated as higher-order tensors that are *N*-way array [20]. The order of a tensor is equal to the number of ways, which are known as modes. GND-PCA directly analyzes the *N*-way array on the sub-mode spaces. This step not only retains the structure of the data, but also overcomes the generalization problem that occurs when the dimension of the unfolded data is much larger than the number of samples.

The bases are calculated on each mode subspace in order to provide a compact representation of the tensors. An iteration algorithm is utilized to obtain the optimal base matrices for each mode. Next, the core tensor containing all principal components is attained with the optimal bases. In this paper, we denote scalars by lower-case letters (*x*, *y*, …), vectors (one-way array) by boldface letters (**x**, **y**, …), matrices (two-way array) by boldface capital letters (**X**, **Y**, …), and third-order tensor (three-way array) by calligraphic capital letters (X,Y,…).

GND-PCA was originally proposed for the construction of statistical appearance models. According to the intrinsic algorithm that lower-rank tensors are found to approximate the original tensors with more compact and meaningful form, we can utilize GND-PCA to retain useful information while suppressing redundancy, that is, image denoising. The next section will introduce our proposed noise reduction method in detail.

## Methodology

**Figure 1:**
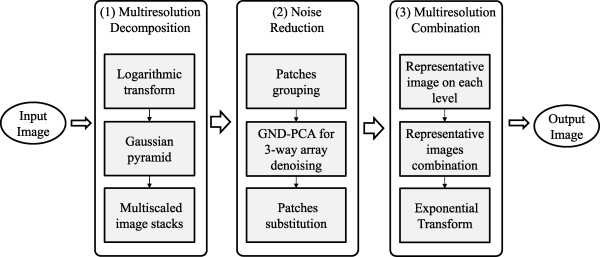
Flowchart of the proposed MR-GND-PCA.

Motivated in part by image pyramids and GND-PCA, we propose a novel ultrasound image denoising method, called multiresolution Generalized *N* Dimension PCA (MR-GND-PCA). Figure 1 shows the proposed MR-GND-PCA algorithm with its three stages.

(1) In the multiresolution decomposition stage, logarithmical transformation is first used to convert the multiplicative speckle noise to an additive noise. Then, a Gaussian pyramid for the noisy image is built using the multiresolution scheme. Multiscale image stacks on each level are built for each Gaussian pyramid.

(2) In the noise reduction stage, similar patches (two-way array) are extracted in each stack and grouped into several categories (three-way array). GND-PCA is directly utilized for three-way arrays to suppress the noise. The denoised patches are put back in the original places to obtain clean multiscale image stacks.

(3) In the multiresolution combination stage, the representative image on each level can be accessed by image averaging. The combination of representative images followed by exponential transformation and sharpen procedure will yield the noise reduction ultrasound image.

The above stages will be formulated in detail in the following subsection.

### Multiresulution decomposition

A reliable model of speckle noise is presented in [21,22]

(1) $$g^{\mathrm{or}}\left(x,y\right)=S^{\mathrm{or}}\left(x,y\right)\eta_{\mathrm{mu}}^{\mathrm{or}}\left(x,y\right)+\eta_{\mathrm{ad}}^{\mathrm{or}}\left(x,y\right)$$

where *S*^*or*^(*x*, *y*) is a noise-free original gray-valued image, *g*^*or*^(*x*, *y*) is an observed noisy gray-valued image, $\eta_{\mathrm{mu}}^{\mathrm{or}}\left(x,y\right)$ is multiplicative noise (coherent interfering), $\eta_{\mathrm{ad}}^{\mathrm{or}}\left(x,y\right)$ is additive noise (such as sensor noise), and *x* and *y* are variables of spatial locations, (*x*, *y*) ∝ **R**^2^. We could approximate the Eq. 1 as Eq. 2, since the effect of additive noise is much smaller than that of multiplicative noise.

(2) $$g^{\mathrm{or}}\left(x,y\right)\approx S^{\mathrm{or}}\left(x,y\right)\eta_{\mathrm{mu}}^{\mathrm{or}}\left(x,y\right)$$

Logarithmic transformation can be executed on both sides of Eq. 2 to separate the noise from the original image.

(3) $$\log\left(g^{\mathrm{or}}\left(x,y\right)\right)\approx \log\left(S^{\mathrm{or}}\left(x,y\right)\right)+\log\left(\eta_{\mathrm{mu}}^{\mathrm{or}}\left(x,y\right)\right)$$

We simplify Eq. 3 as Eq. 4 without modifying the definition:

(4) $$g\left(x,y\right)\approx S\left(x,y\right)+\eta \left(x,y\right)$$

Therefore, multiplicative speckle noise is converted to additive noise with logarithmical transformation. This step is more reasonable for the proposed MR-GND-PCA as applied to ultrasound image denoising.

The Gaussian pyramid for the noisy image in the logarithmic space and the multiscale image stacks on each pyramid level are built successively because only the possible scale-space kernel is a Gaussian function [23]. The scale space of the image *g* is produced from the convolution of variable-scale Gaussian filters *f*(*σ*, (*x*, *y*)) with the input image:

(5) $$T\left(g\right)=f\left(\sigma ,\left(x,y\right)\right)*g\left(x,y\right)$$

where “*” is the convolution operation in *x* and *y*, and *σ* denotes the scale parameter, and

(6) $$f\left(\sigma ,\left(x,y\right)\right)=\frac{1}{2\pi \sigma^{2}}e^{-\left(x^{2}+y^{2}\right)/2\sigma^{2}}$$

**Figure 2:**
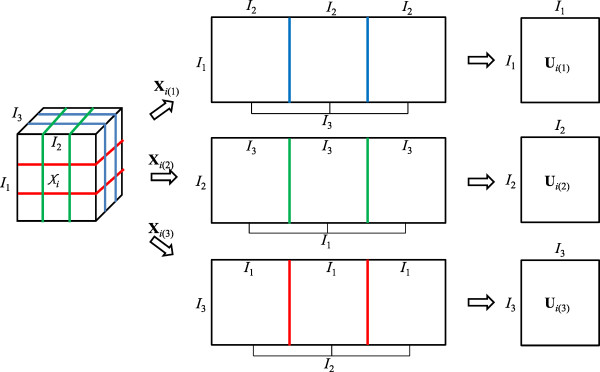
The algorithm of MR-GND-PCA.

In our paper, an associated Gaussian pyramid *G*_*a*,0_, 0 ≤ *a* ≤ *A* is composed of a set of different resolutions of *g*. Here, *A* + 1 is the number of total levels and *G*_0,0_ = *g. G*_*a*,0_ are created by applying the Gaussian filter and successive downsampling. Both operations are combined in the “Reduce” operation, and shown on the first line in Figure 2,

(7) $$\mathrm{Reduce}\left(G_{a,0}\right)=\left(2↓\right)\left(f_{1}*G_{a,0}\right)$$

where *f*_1_ denotes a linear low-pass Gaussian filter, and (2 ↓) denotes downsampling that discards every data element with an odd index and is applied to each dimension of the image.

Multiscale image stacks on each pyramid level are subsequently constructed. Assume *G*_*a*,0_ as the basic image of the *a*^th^ stack at each level of the Gaussian pyramid. *G*_*a*,*b*_, 0 ≤ *b* ≤ *B* are a series of different corresponding scales of *G*_*a*,0_; *B* + 1 is the number of scales on each level. The multiscale image stacks *G*_*a*,*b*_, 0 ≤ *b* ≤ *B* are created by applying the low-pass Gaussian filter *f*_2_. Thus, we have

(8) $$G_{a,b}=\left\{\begin{array}{l} G_{a,0},\mathrm{Num}=0\\ \left(\mathrm{Num}\right)f_{2}*G_{a,0},1\leq \mathrm{Num}\leq B \end{array}\right)$$

where (Num) denotes the number of *f*_2_ convolutions with *G*_*a*,0_. Both Gaussian filters *f*_1_ and *f*_2_ are defined in 5 × 5 sliding windows.

### Noise reduction

GND-PCA as a multilinear subspace method is utilized for denoising the images of each stack on the pyramid level to reduce noise for the entire image. Take the first stack, *G*_0,*b*_, 0 ≤ *b* ≤ *B*, for example. *Q* overlapping dense patches, $X_{q}^{\left(0\right)}\in R^{I_{1}\times I_{2}},q=1,2,\ldots ,Q$, are extracted from all images in this stack. Here, *I*_1_ × *I*_2_ is the size of the patch, and the superiors “(0)” denotes that all the extracted patches are in the first stack. Organizing the patches into sensible groupings is a fundamental process for GND-PCA denoising. Cluster analysis, which consists of hard and soft clustering, implements groupings according to measured intrinsic characteristics or similarities. Loosely speaking, hard clustering assigns the data point to one and only one cluster. In contrast, soft clustering assigns the data point to all clusters with different weights whose sum equals one. Clustering is unsupervised learning due to the absence of category information. Despite the fact that thousands of clustering algorithms have been published, we adopt the most popular and simple clustering algorithm, *K*-means [24], for our research. As the discussion of diversified clustering algorithms is beyond our scope, please refer to [25] for more details on data clustering.

The advantage of containing the original measured quantities and easy implementation allows for pixel intensity values instead of patch features to be employed for clustering. The overlapping dense patches on the first stack are unfolded as the set of Q (*I*_1_ × *I*_2_) -dimensional points $\left[{\hat{x}}_{1}^{\left(0\right)},{\hat{x}}_{2}^{\left(0\right)},\ldots ,{\hat{x}}_{Q}^{\left(0\right)}\right],q=1,2,\ldots ,Q$ that will be clustered into *K* clusters. Nevertheless, massive noise existing in the patches decreases the reliability of the characteristic structure and eliminates the performance of the clustering algorithm. In consideration of computational effectiveness, (*I*_1_ × *I*_2_) -dimensional points are projected into the lower dimensional space by PCA to characterize the intrinsic variation of the data. After projection, (*I*_1_ × *I*_2_) -dimensional points $\left[{\hat{x}}_{1}^{\left(0\right)},{\hat{x}}_{2}^{\left(0\right)},\ldots ,{\hat{x}}_{Q}^{\left(0\right)}\right]$ is converted into *J* dimensional points $\left[x_{1}^{\left(0\right)},x_{2}^{\left(0\right)},\ldots ,x_{Q}^{\left(0\right)}\right]$.

Assume $C^{\left(0\right)}=\left[c_{1}^{\left(0\right)},c_{2}^{\left(0\right)},\ldots ,c_{k}^{\left(0\right)},\ldots ,c_{K}^{\left(0\right)}\right],k=1,2,\ldots ,K$ denotes *K* clusters and $\mu_{k}^{\left(0\right)}$ denotes the mean of cluster *c*_*k*_. A partition of the patches can be found by minimizing the squared error between the grouping means and the points:

(9) $$\mathrm{cluster}\left(C^{\left(0\right)}\right)=\underset{\mu_{k}^{\left(0\right)}}{\arg\min}\sum_{k=1}^{K}\sum_{x_{q}^{\left(0\right)}\in c_{k}^{\left(0\right)}}{\left\|x_{q}^{\left(0\right)}‒\mu_{k}^{\left(0\right)}\right\|}^{2}$$

After clustering in one multiscale image stack, the corresponding patches that exhibit high correlation are grouped into *K* three-way arrays $X_{i}^{\left(0\right)}\in R^{I_{1}\times I_{2}\times I_{3}},i=1,2,\ldots ,K$, with *I*_3_ denoting the number of similar images in the group. Three-way arrays are processed precisely by GND-PCA to suppress noise without destroying the internal connection of patch similarities.

**Figure 3:**
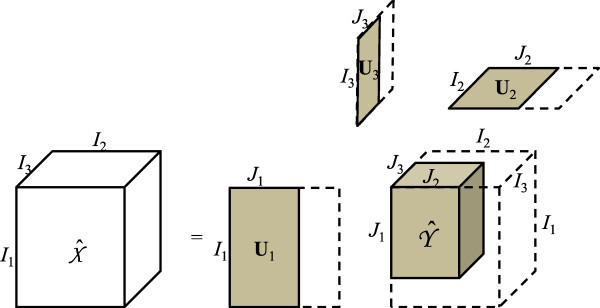
Mode matricization of a three-way array and calculation of eigenvector matrices.

**Figure 4:**
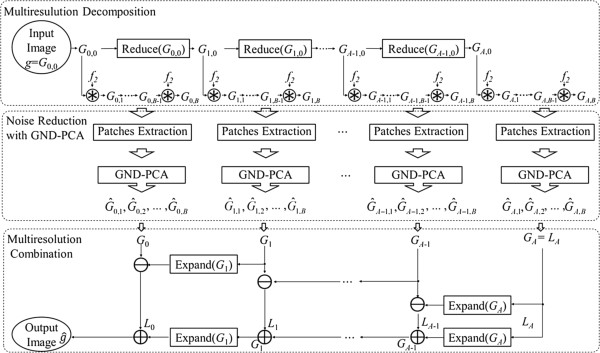
Dimension reduction of GND-PCA with a three-way array.

A three-way array $X_{i}^{\left(0\right)}\in R^{I_{1}\times I_{2}\times I_{3}},i=1,2,\ldots ,K$ can be unfolded into three matrices along each mode space and represented by $X_{i\left(1\right)}^{\left(0\right)}\in R^{I_{1}\times \left(I_{2}\cdot I_{3}\right)}$, $X_{i\left(2\right)}^{\left(0\right)}\in R^{I_{2}\times \left(I_{3}\cdot I_{1}\right)}$, and $X_{i\left(3\right)}^{\left(0\right)}\in R^{I_{3}\times \left(I_{1}\cdot I_{2}\right)},i=1,2,\ldots ,K$. The unfolding procedures, called mode matricization of a third-order tensor, are visualized in Figure 3. For each unfolded matrix, the eigenvector matrix $U_{i\left(n\right)}^{\left(0\right)}=\left[u_{i\left(n\right)1}^{\left(0\right)},u_{i\left(n\right)2}^{\left(0\right)},\cdots ,u_{i\left(n\right)D_{i\left(n\right)}}^{\left(0\right)}\right]$ associated with the first $D_{i\left(n\right)}^{\left(0\right)}$ largest eigenvalues (number of mode-subspace bases) can be calculated from the covariance matrix $C_{X_{i\left(n\right)}^{\left(0\right)}}=E\left\{X_{i\left(n\right)}^{\left(0\right)}{X_{i\left(n\right)}^{\left(0\right)}}^{T}\right\}$, where, *n* = 1, 2, 3. Generally, the number of *D*_*i*(*n*)_ are decided by the experience and application. A simple determination procedure proposed in [26] can also be used. In each mode, this method decides the dimension by the variation ratio to be kept by eigenvalues. The order of the eigenvectors, $u_{i\left(n\right)1}^{\left(0\right)},u_{i\left(n\right)2}^{\left(0\right)},\cdots ,u_{i\left(n\right)D_{i\left(n\right)}}^{\left(0\right)}$, satisfies the corresponding eigenvalues $\lambda_{i\left(n\right)1}^{\left(0\right)}\geq \lambda_{i\left(n\right)2}^{\left(0\right)}\geq \ldots \geq \lambda_{i\left(n\right)D_{i\left(n\right)}}^{\left(0\right)}$. Dimension reduction is completed in each mode for denoising, and thus, the calculated $U_{i\left(n\right)}^{\left(0\right)}$ are not the optimal bases for the entire three-way array. The optimal bases for the three-way array and denoised patches can be obtained by utilizing the above $U_{i\left(n\right)}^{\left(0\right)}$ as the initialization. Then, an iteration algorithm, which calculates one matrix if the others are fixed, is used to determine all optimal matrices to satisfy the following energy function:

(10) $$e=\underset{U_{i\left(n\right)}^{\left(0\right)},n=1,2,3}{\arg\min}{\left\|X_{i}^{\left(0\right)}‒\left(Y_{i}^{\left(0\right)}\times_{1}U_{i\left(1\right)}^{\left(0\right)}\times_{2}U_{i\left(2\right)}^{\left(0\right)}\times_{3}U_{i\left(3\right)}^{\left(0\right)}\right)\right\|}^{2}$$

where $Y_{i}^{\left(0\right)}\in R^{D_{i\left(1\right)}^{\left(0\right)}\times D_{i\left(2\right)}^{\left(0\right)}\times I_{3}}$ are core tensors. Note that the dimension of the third mode is retained without reduction. The reconstructed three-way array shown in Eq. 11 is the denoised grouping. Figure 4 illustrates the dimension reduction of GND-PCA with a three-way array.

(11) $${\hat{X}}_{i}^{\left(0\right)}={\hat{Y}}_{i}^{\left(0\right)}\times_{1}{\hat{U}}_{i\left(1\right)}^{\left(0\right)}\times_{2}{\hat{U}}_{i\left(2\right)}^{\left(0\right)}\times_{3}{\hat{U}}_{i\left(3\right)}^{\left(0\right)}$$

After all the three-way arrays on the same level are denoised with GND-PCA, we place them back into their original positions. With the overlapping patches extracted, more than one patch estimation in the denoised three-way arrays can be located at exactly the same coordinate. An accumulation is performed by averaging the overlapping pixel positions. Take the example on *G*_0,0_, which denotes the first level image with no Gaussian filter. The denoised estimation ${\hat{G}}_{0,0}$ of *G*_0,0_ is computed by an average of the patch estimates ${\hat{Y}}_{p}^{\left(0\right)}\in R^{I_{1}\times I_{2}},p=1,2,\ldots ,P$ in the first stack. Here, ${\hat{Y}}_{p}^{\left(0\right)}\in {\hat{Y}}_{q}^{\left(0\right)},P\leq Q$. We have

(12) $${\hat{G}}_{0,0}=\frac{\sum_{p\in \left[1,P\right]}\sum_{x_{R}}{\hat{Y}}_{p}^{\left(0\right),x_{R}}}{\sum_{p\in \left[1,P\right]}\sum_{x_{R}}E^{x_{R}}}$$

where **E** has the same size as ${\hat{Y}}_{p}^{\left(0\right)}$ and all elements of **E** are one, and *x*_*R*_ is the coordinate iterated in patches. The denoised estimations for all stacks are denoted by ${\hat{G}}_{a,b},0\leq a\leq A,0\leq b\leq B$.

### Multiresolution combination

All images on the same stack are added up to become one representative image, as shown in Eq. 13.

(13) $$G_{a}=\frac{1}{B+1}\sum_{b=0}^{B}{\hat{G}}_{a,b}$$

The denoised image is obtained by combining the representative images *G*_*a*_, *a* = 0, 1, 2, …, *A* at each level. The corresponding pyramids with levels *L*_*a*_ contain the differences of high-frequency components between *G*_*a*_ and *G*_*a* + 1_. Differences *G*_*a*_ − *G*_*a* + 1_, *a* = 0, 1, …, *A* − 1 cannot be calculated directly because *G*_*a* + 1_ contains fewer samples than *G*_*a*_. For this reason, the number of samples in *G*_*a* + 1_ is increased to match *G*_*a*_ by interpolating the missing samples and the interpolation filter. Both operations are combined in the “Expand” operation,

(14) $$\mathrm{Expand}\left(G_{a}\right)=f_{3}*\left[\left(2↑\right)G_{a}\right]$$

where (2 ↑) denotes upsampling by inserting a zero between adjacent data elements and *f*_3_ denotes the linear interpolation filter. The output image $\hat{g}$ can be reconstructed from *L*_*a*_ by,

(15) $${\hat{G}}_{a}=\left\{\begin{array}{l} L_{a}:a=A\\ L_{a}+\mathrm{Expand}\left(G_{a+1}\right):0\leq a\leq A-1 \end{array}\right)$$

and then,

(16) $$\hat{g}={\hat{G}}_{0}$$

In the reconstructed process, the low-frequency information of *G*_*a*_ is discarded and replaced by *G*_*a* + 1_. Finally, the de-speckled image in log-domain is converted into spatial domain through exponential transformation. The complete MR-GND-PCA method is shown as a diagrammatic sketch in Figure 2.

## Experiments and results

The proposed MR-GND-PCA was used as an application to denoise an ultrasound image corrupted by speckle noise. The quantitative performance of the proposed method was investigated by using three different kinds of images, including a synthetic image with artificial speckle noise, phantom data, and a common carotid artery scanned by commercial ultrasonic scanners.

### Synthetic image investigation

**Figure 5:**
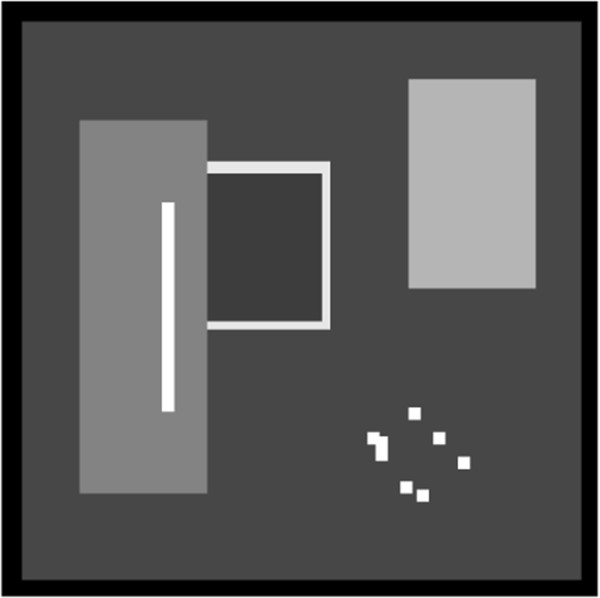
Original purely synthetic image.

The efficiency of the proposed MR-GND-PCA was tested by using a generated noisy image with artificial speckle noise. Equation 2 shows that we degraded the original test image by multiplying it with unit mean random fields to obtain the test speckle image. The spatially correlated speckle noise was produced by low-pass filtering a complex Gaussian random field and taking the magnitude of the filtered output [27]. Low-pass filtering was performed by averaging the complex values of the size three sliding window. This short-term correlation allowed the noise correlation to taper gradually to zero, as well as the correlation length of the speckle to be controlled by appropriately setting the size of the kernel used to introduce correlation to the underlying Gaussian noise. The size of the synthetic image is 141 × 141. Two distinct levels of speckle noise were generated by changing the variance of the underlying complex Gaussian random field. The purely synthetic image shown in Figure 5 is invoked as a reference noise-free image and is available at [34]. The image is comprised of regions with uniform intensity, sharp edges, and strong scatters.

The performance of the proposed MR-GND-PCA method was compared with traditional despeckling methods (Median filter, Fourier ideal filtering, Fourier Butterworth filter, Wavelet filter) as well as state-of-the-art algorithms (Bilateral filter [28], KSVD [12], Non-local means [29], BM3DPCA [13], PGPCA [14], LPGPCA [15]). All despeckling methods were implemented on the images with high, middle and low levels of speckle noise.

We used four well-known metrics as quantitative performance measures to assess quality image, including mean-square error (MSE), signal-to-noise ratio (SNR), peak signal-to-noise ratio (PSNR), and the structural similarity (SSIM). MSE evaluates the noise variance and is given by

(17) $$\mathrm{MSE}=\frac{1}{\mathrm{MN}}\sum_{x=1}^{M}\sum_{y=1}^{N}{\left|\hat{g}\left(x,y\right)-S\left(x,y\right)\right|}^{2}$$

where $\hat{g}\left(x,y\right)$ is the denoised image and *S*(*x*, *y*) is the purely synthetic image. SNR is defined as

(18) $$\mathrm{SNR}=10\cdot \log_{10}\frac{P_{s}}{\mathrm{MSE}}$$

where *P*_*s*_ is the variance of the noise-free reference image as

(19) $$P_{s}=\frac{1}{\mathrm{MN}}\sum_{x=1}^{M}\sum_{y=1}^{N}{\left(\hat{g}\left(x,y\right)\right)}^{2}$$

PSNR is defined by

(20) $$\mathrm{PSNR}=10\cdot \log_{10}\frac{N_{\text{max}}}{\mathrm{MSE}}$$

where *N*_max_ is the maximum fluctuation in the input image.

And SSIM is given by

(21) $$\mathrm{SSIM}=\frac{\left(2\mu_{\hat{g}\left(x,y\right)}\mu_{S\left(x,y\right)}+C_{1}\right)\left(2\sigma_{\hat{g}\left(x,y\right)S\left(x,y\right)}+C_{2}\right)}{\left(\mu_{\hat{g}\left(x,y\right)}^{2}+\mu_{S\left(x,y\right)}^{2}+C_{1}\right)\left(\sigma_{\hat{g}\left(x,y\right)}^{2}+\sigma_{S\left(x,y\right)}^{2}+C_{2}\right)}$$

where $\mu_{\hat{g}\left(x,y\right)}$, *μ*_*S*(*x*,*y*)_, $\sigma_{\hat{g}\left(x,y\right)}$, *σ*_*S*(*x*,*y*)_, and $\sigma_{\hat{g}\left(x,y\right)S\left(x,y\right)}$ are the mean of $\hat{g}\left(x,y\right)$, the mean of *S*(*x*, *y*), the variance of $\hat{g}\left(x,y\right)$, the variance of *S*(*x*, *y*), and the covariance of $\hat{g}\left(x,y\right)$ and *S*(*x*, *y*), respectively; *C*_1_ and *C*_2_ are small constants.

#### Multiple parameter selection

**Figure 6:**
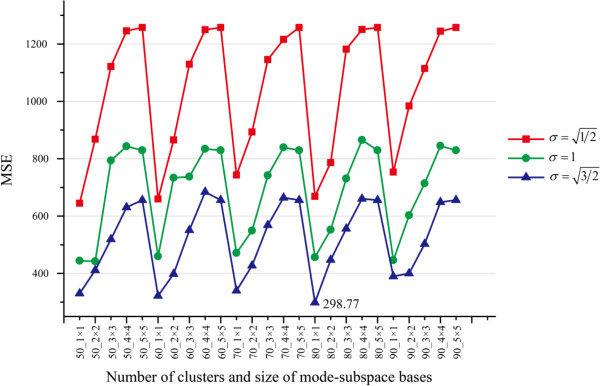
Variation trends of MSE with different number of clusters, number of mode-subspace bases and Gaussian scales.

**Figure 7:**
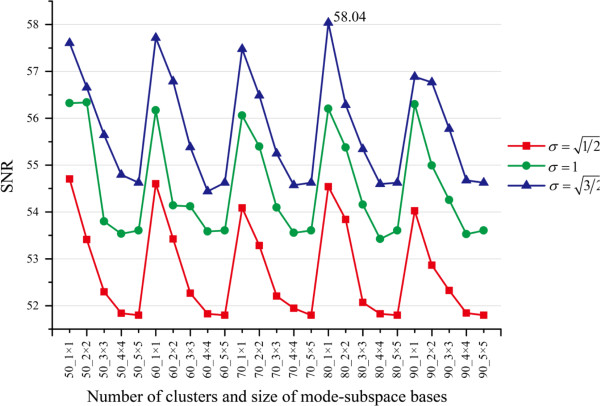
Variation trends of SSIM with different number of clusters, number of mode-subspace bases and Gaussian scales.

**Figure 8:**
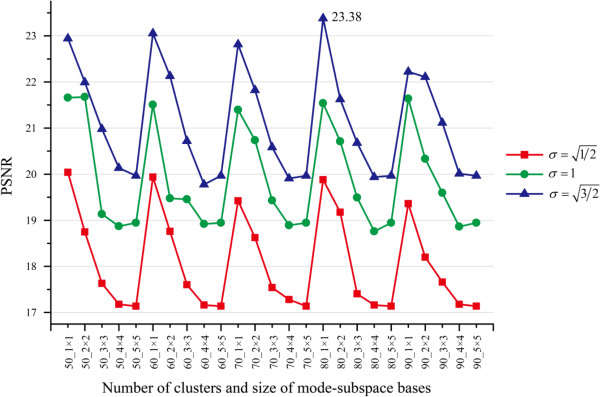
Variation trends of SNR with different number of clusters, number of mode-subspace bases and Gaussian scales.

**Figure 9:**
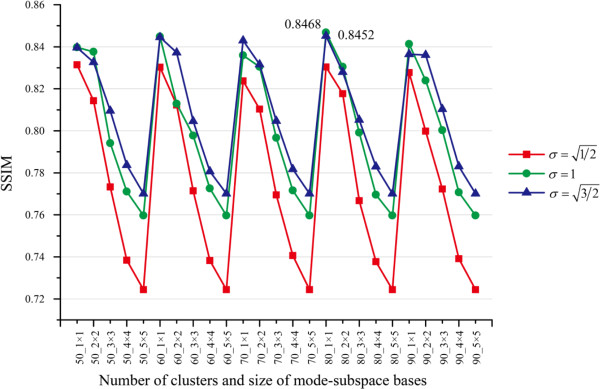
Variation trends of PSNR with different number of clusters, number of mode-subspace bases and Gaussian scales.

In MR-GND-PCA, the parameters: number of mode-subspace bases *D*_*i*(1)_ × *D*_*i*(2)_ , number of clusters (*K*), and the Gaussian scale (σ) are crucial for restoration results. In order to determine the best parameters of MR-GND-PCA, we investigate the variation trends of MSE, SNR, PSNR and SSIM when *D*_*i*(1)_, *D*_*i*(2)_ ∈ [1, 5], *K* = 50 ~ 90, and $\sigma =\sqrt{1/2},1,\sqrt{3/2}$. The comparison results are illustrated in Figures 6, 7, 8 and 9. It is clear in Figures 6, 7 and 8 that the lowest MSE, the highest SNR and PSNR can be obtained when *D*_*i*(1)_ × *D*_*i*(2)_ = 1 × 1, K = 80 and $\sigma =\sqrt{3/2}$, which are also considered as the best parameters in the following experiments, though SSIM is a little bit higher with *σ* = 1 than that with $\sigma =\sqrt{3/2}$ in Figure 9.

#### Comparison of MR-PCA and MR-GND-PCA

**Figure 10:**
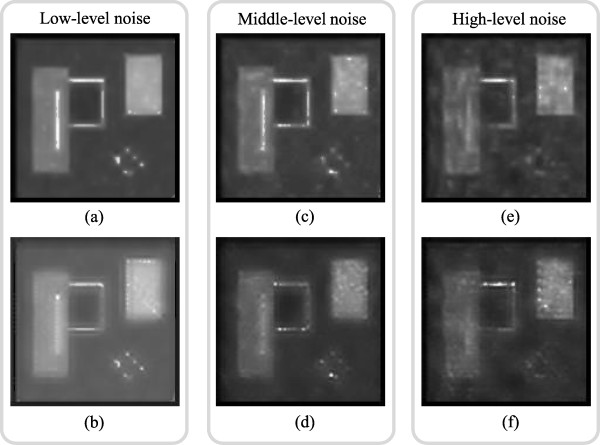
Restoration results for low-, middle-, and high-level noise with MR-GND-PCA and MR-PCA: (a) Restoration result for low-level noise with MR-GND-PCA (MSE = 298.77, SNR = 58.04, PSNR = 23.38, SSIM = 0.8452) (b) Restoration result for low-level noise with MR-PCA (MSE = 820.8849, SNR = 53.65, PSNR = 18.99, SSIM = 0.7359), (c) Restoration result for middle-level noise with MR-GND-PCA (MSE = 532.83, SNR = 55.53, PSNR = 20.86, SSIM = 0.7738), (d) Restoration result for middle-level noise with MR-PCA(MSE = 1422.50, SNR = 51.26, PSNR = 16.60, SSIM = 0.6606), (e) Restoration result for high-level noise with MR-GND-PCA (MSE = 985.93, SNR = 52.85, PSNR = 18.19, SSIM = 0.6775), (f) Restoration result for high-level noise with MR-PCA (MSE = 1423.3, SNR = 51.2604, PSNR = 16.60, SSIM = 0.6484).

In order to illustrate the effectiveness of GND-PCA for image denoising, we substitute GND-PCA for PCA in the proposed MR-GND-PCA framework, which is called MR-PCA. The restoration results for low-, middle-, and high-level noise are shown in Figure 10. All the parameters in MR-PCA are set completely same as those in MR-GND-PCA. The first row ((a), (c) and (e)) presents the restoration results obtained with MR-GND-PCA, and the second row ((b), (d) and (f)) presents the restoration results obtained with MR-PCA. The calculated MSE, SNR, PSNR and SSIM related to the original image are also given in the captions below the figure. We can see that MR-GND-PCA gives better overall performance in terms of both noise suppression and detail preservation. However, MR-PCA produces artifacts around the edges of each objects, which is a potential influence of the accurate diagnosis.

#### Comparison of multiple denoising methods

The values of MSE, SNR, PSNR and SSIM obtained for the corresponding test image with all the methods are summed up in Tables 1, 2 and 3.

**Table 1:** Metrics obtained when applying denoising methods to the image with low-level of speckle noise

**Denoising methods**	**MSE**	**SNR**	**PSNR**	**SSIM**
The source image	1766.6	50.32	15.66	0.4679
Bilateral filter	388.63	57.01	22.24	0.7465
KSVD	1046.8	52.70	17.93	0.8208
NLM	1046.2	52.70	17.93	0.8056
Fourier ideal filtering	849.51	53.61	18.84	0.5818
Fourier Butterworth	810.66	53.81	19.04	0.6451
Median filter	741.61	54.20	19.43	0.6678
Wavelet filter	1039.7	52.73	17.96	0.5604
BM3DPCA	936.26	53.19	18.42	0.8359
PGPCA	457.91	56.29	21.52	0.8415
LPGPCA	723.43	54.31	19.54	0.8405
MR-GND-PCA	298.77	58.04	23.38	0.8452

**Table 2:** Metrics obtained when applying denoising methods to the image with middle-level speckle noise

**Denoising methods**	**MSE**	**SNR**	**PSNR**	**SSIM**
The source image	3115.3	47.97	13.20	0.2624
Bilateral filter	574.56	55.31	20.54	0.6646
KSVD	2136.6	49.60	14.83	0.6086
NLM	1495.00	51.15	16.38	0.6447
Fourier ideal filtering	602.14	55.10	20.33	0.4763
Fourier Butterworth	623.03	54.96	20.19	0.5384
Median filter	649.68	54.77	20.00	0.5296
Wavelet filter	1710.9	50.57	15.80	0.3978
BM3DPCA	575.26	55.30	20.53	0.6883
PGPCA	704.16	54.42	19.65	0.7377
LPGPCA	770.55	54.03	19.26	0.7472
MR-GND-PCA	532.83	55.53	20.86	0.7738

**Table 3:** Metrics obtained when applying denoising methods to the image with high-level speckle noise

**Denoising methods**	**MSE**	**SNR**	**PSNR**	**SSIM**
The source image	3685.6	47.24	12.47	0.2294
Bilateral filter	1723.8	50.54	15.77	0.5777
KSVD	2766.9	48.48	13.71	0.5323
NLM	2929.5	48.23	13.46	0.5178
Fourier ideal filtering	1319.8	51.70	16.93	0.3540
Fourier Butterworth	1078.1	52.57	17.80	0.4074
Median filter	1228.6	52.01	17.24	0.3903
Wavelet Filter	2636.7	48.69	13.92	0.3199
BM3DPCA	1720.6	50.54	15.77	0.5063
PGPCA	1516.9	51.09	16.32	0.6634
LPGPCA	1542.6	51.02	16.25	0.6078
MR-GND-PCA	985.93	52.85	18.19	0.6775

**Figure 11:**
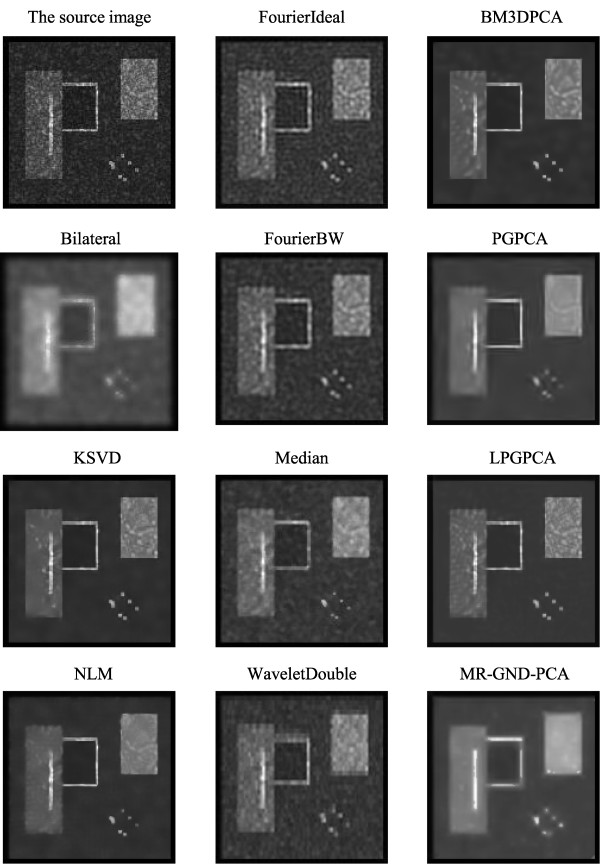
Images after applying different denoising methods for purely synthetic image with low-level speckle noise.

**Figure 12:**
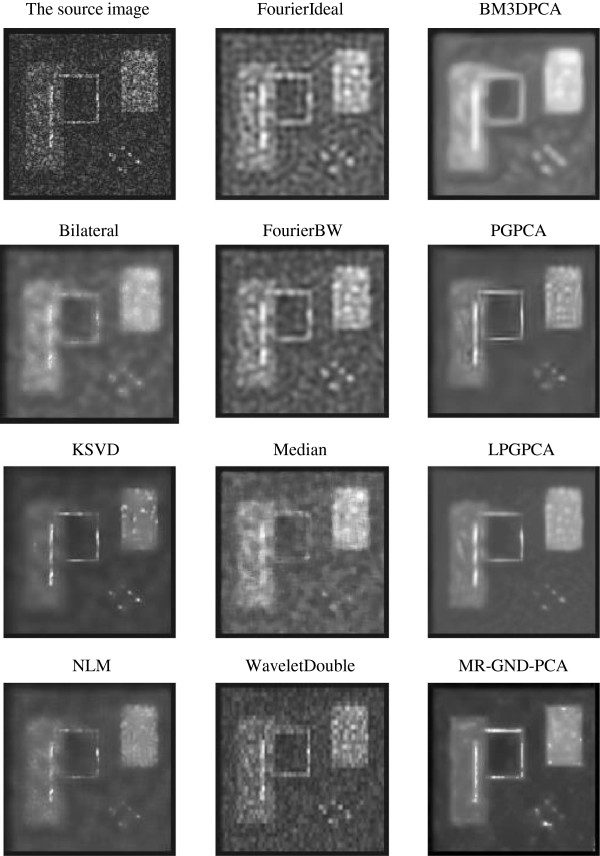
Images after the application of different denoising methods for the purely synthetic image with middle-level speckle noise.

**Figure 13:**
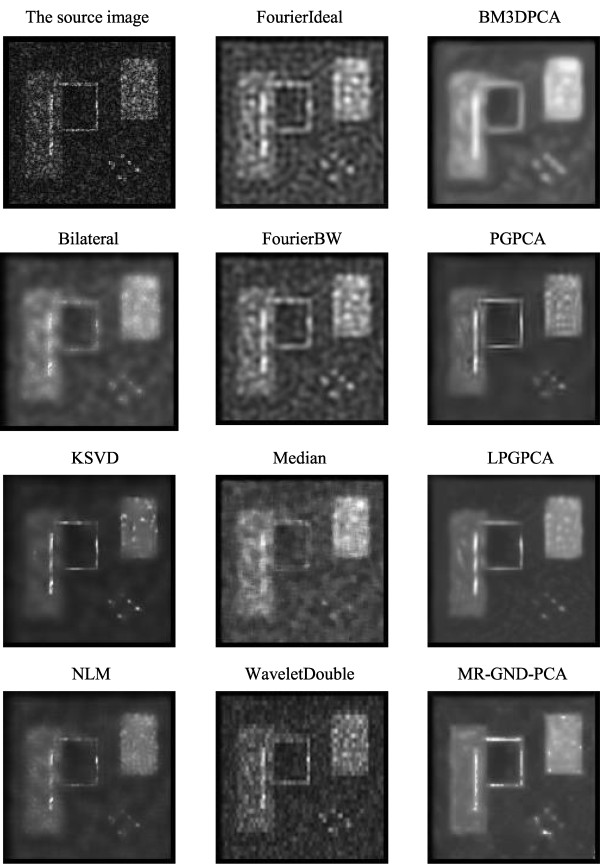
Images after the application of different denoising methods for the purely synthetic image with high-level speckle noise.

Tables 1, 2 and 3 clearly show that the proposed MR-GND-PCA can obtain the lowest MSE, as well as the highest SNR and PSNR. Moreover, the SSIMs of the proposed method are nearest to one among all the denoising filters. Thus, the MR-GND-PCA is an effective denoising method for reducing the noise as well as preserving the details. Comparisons of Figures 11, 12 and 13 show that the proposed MR-GND-PCA can obtain more impressive denoising results for the image with high level noise. Although there are still some artifacts in the smoothed images with all the denoising methods, such as upper right object, the proposed MR-GND-PCA is the most reliable process algorithm because of suppressing the majority noise keeping the details in the source image. The result confirms that the multiresolution created for GND-PCA performance is an appropriate combination for ultrasound images with massive noise compare to the state-of-art methods.

### Phantom data validation

**Figure 14:**
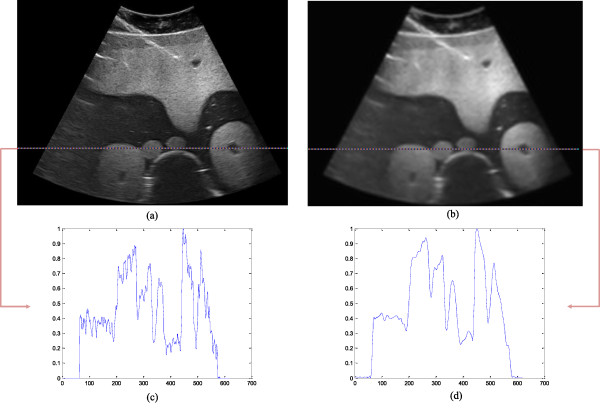
Results on phantom data: (a) original image, (b) denoised image, (c) profile row phantom data, (d) profile row denoised image.

Phantom data called CIRS MODEL 057A are available at *http://www.cirsinc.com/*. The B-scan image of these phantom data was obtained from a commercial ultrasonic scanner with a 3.5 MHz phased array probe. The 470 × 620 regions of interest of the original and processed images are shown in Figures 14(a) and (b). The highlighted lines in Figures 14(a) and (b) indicate the 340th rows in the images. The pixel values in these rows from the original and denoised images are compared in Figures 14(c) and (d) respectively. Our proposed method suppresses speckle noise effectively. Most of the stray mottles in the original image are smoothed.

### Clinical data verification

**Figure 15:**
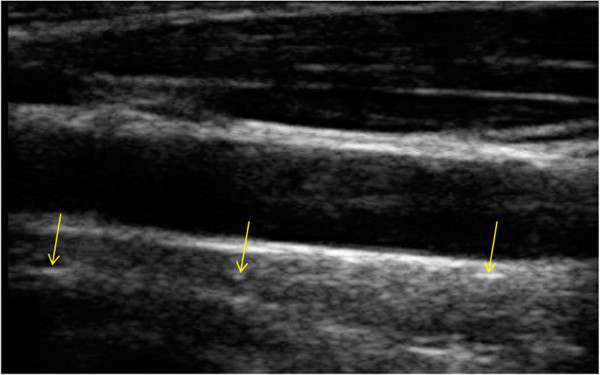
**Original CCA image.** The three yellow arrows point to the focus locations.

**Figure 16:**
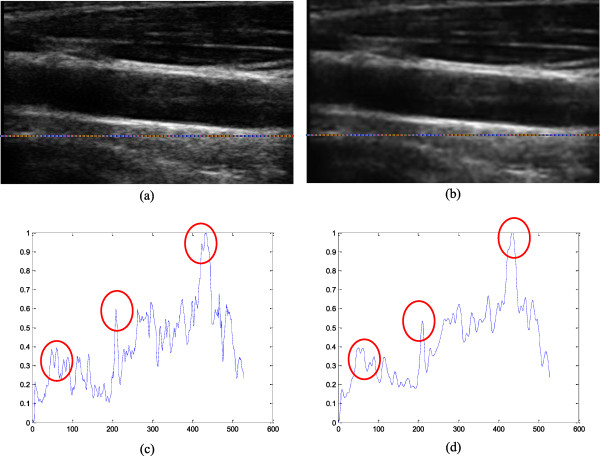
Results on CCA image: (a) original image, (b) denoised image, (c) profile row CCA image, (d) profile row denoised image.

Clinical images of common carotid artery (CCA) are available for free on the Signal Processing Laboratory website. The B-scans were acquired with a Sonix OP ultrasound scanner with a different set-up of depth, gain, time gain compensation (TGC) curve, and different linear array transducers. Figure 15 shows one of the CCA images with size of 330 × 528, in which three highlighted areas marked by the yellow arrows can be fully identified and preserved. Figure 16 illustrates the results processed before and after using MR-GND-PCA. The highlighted areas in Figures 16(c) and (d) show that the denoised image in Figure 16(b) displays significant improvement and recovers discontinuities well.

## Discussion and conclusions

In this paper, we introduced MR-GND-PCA for noise suppression in ultrasound images. Our approach consists of three stages. First, after the ultrasound image is converted by logarithmical transformation, the Gaussian pyramid with multiscale image stacks on each level is built. Second, similar patches are grouped to be directly denoised by GND-PCA in the same pyramid level and then placed on the original location to form clean pyramid levels. Third, the representative images obtained from each level are combined to obtain the noise reduction ultrasound image.

The first and third stages create a multiresolution framework that utilizes all frequency bands. Downscaling has a denoising effect that contributes to better denoising. The second stage uses GND-PCA to maintain the structure of the data and to consider the correlation of all modes of the proposed data.

Experiments were carried out on synthetic images with three distinctive level speckles and two clinical images. Quantitative measures were used to compare 11 denoising filters for the synthetic images. Experimental results show that our proposed method achieved the lowest noise interference. Our method is also robust for the image with a much higher level of speckle noise. For clinical images, the results show that MR-GND-PCA can reduce speckle and preserve resolvable details. Further work on speeding up the method and enhancing the edge of the objects will be pursued in the future.

## Competing interests

The authors declare that they have no competing interests.

## Authors’ contributions

ADN suggested the algorithm for images denoising, implemented it and drafted the manuscript. YJ conceived of the study, and participated in its design and coordination and helped to draft the manuscript. CY performed the acquisition of the ultrasound images and expressed opinions on the evaluation metric of the denoised results. CWJ and FJF participated in the design of the study, drew a part of figures and performed the image analysis. WYT helped to draft the manuscript and expressed opinions on the overall framework of the manuscript. All authors have read and approved the final manuscript.
